# A high performance wearable strain sensor with advanced thermal management for motion monitoring

**DOI:** 10.1038/s41467-020-17301-6

**Published:** 2020-07-15

**Authors:** Cenxiao Tan, Zhigang Dong, Yehua Li, Haiguang Zhao, Xingyi Huang, Zhaocai Zhou, Jin-Wu Jiang, Yun-Ze Long, Pingkai Jiang, Tong-Yi Zhang, Bin Sun

**Affiliations:** 1 College of Physics; University-Industry Joint Center for Ocean Observation and Broadband Communication; State Key Laboratory of Bio-Fibers and Eco-Textiles, Qingdao University, 266071 Qingdao, P. R. China; 2 School of Physical Education, Qingdao University, 266071 Qingdao, P. R. China; 3 State Key Laboratory of Genetic Engineering, School of Life Sciences, Fudan University, 200438 Shanghai, P. R. China; 4 Department of Polymer Science and Engineering, Shanghai Key Laboratory of Electrical Insulation and Thermal Aging, Shanghai Jiao Tong University, 200240 Shanghai, P. R. China; 5 Shanghai Institute of Applied Mathematics and Mechanics, Shanghai University, 200444 Shanghai, P. R. China; 6 Materials Genome Institute, Shanghai University, 200444 Shanghai, P. R. China

**Keywords:** Electrical and electronic engineering, Sensors and biosensors, Organic-inorganic nanostructures, Organic-inorganic nanostructures

## Abstract

Resistance change under mechanical stimuli arouses mass operational heat, damaging the performance, lifetime, and reliability of stretchable electronic devices, therefore rapid thermal heat dissipating is necessary. Here we report a stretchable strain sensor with outstanding thermal management. Besides a high stretchability and sensitivity testified by human motion monitoring, as well as long-term durability, an enhanced thermal conductivity from the casted thermoplastic polyurethane-boron nitride nanosheets layer helps rapid heat transmission to the environments, while the porous electrospun fibrous thermoplastic polyurethane membrane leads to thermal insulation. A 32% drop of the real time saturated temperature is achieved. For the first time we in-situ investigated the dynamic operational temperature fluctuation of stretchable electronics under repeating stretching-releasing processes. Finally, cytotoxicity test confirms that the nanofillers are tightly restricted in the nanocomposites, making it harmless to human health. All the results prove it an excellent candidate for the next-generation of wearable devices.

## Abstract (web summary)

Though stretchable strain sensors are attractive for next-generation applications due to their high sensitivity, heat generated in these devices limits their reliability. Here, the authors report boron nitride nanosheet-based stretchable strain sensors with enhanced thermal management.

## Introduction

Wearable and flexible/stretchable strain sensors have attracted great attention nowadays because they can convert mechanical deformations into electrical signals^1,2^, and are widely used in soft robotics^3,4^, human–machine interaction^5^, health-monitoring systems^6,7^, and human motion monitoring and detection^8,9^. Compared with their traditional rigid counterparts that can bear a little strain (less than 5%)^10^, flexible/stretchable strain sensors are able to sustain a larger external strain^11,12^. In particular, stretchable strain sensors can undergo larger deformation than that of the flexible ones, endowing them attractive to measure movements on stretchable and curved surfaces. There are many types of stretchable strain sensors, including resistive sensors, capacitive sensors, piezoelectric sensors, triboelectric sensors, and so on^13,14^. Among them, stretchable resistive strain sensors with high sensitivity are extremely desirable due to their simple structure and facile fabrication process^15,16^. Carbon nanomaterials have intriguing properties, such as outstanding flexibility, large surface–volume ratio, high chemical and thermal stability, and good electrical conductivity^17,18^. Stretchable resistive strain sensors based on graphene^19^, carbon nanotubes (CNTs)^20,21^, etc., have been proposed recently. In order to acquire a high sensitivity, the microstructures of active materials have to change dramatically under subtle mechanical stimuli in the form of resistance. It is worthy to note that the resistance change, particularly large nanocontact resistance between nanofillers^22,23^, will consequently generate a mass of heat, usually been adopted as nanoheaters^24–27^. However, for stretchable electronics, this elevated heat will negatively affect the performance, safety, and reliability of the devices, and even cause them to fail in structure and function. For the purpose of dissipating thermal heat from these devices, materials with enhanced thermal conductivity are strongly recommended^28^. There are publications of nanocomposites based on electrical conductive materials/polymer for thermal conduction^29–32^, yet the exposure of electrical conductive materials of electronics and electrical devices may cause the contamination and corrosion, and even the risk of electric shock. Hence, encapsulation with electrical insulating materials is the necessary prerequisite. However, the low thermal conductivity of commonly used packaging materials, such as PDMS and epoxy, will hinder the heat dissipation of the electronics. Therefore, electrical insulating materials with high thermal conductivity are of significance for the packaging of modern electronics. On the other hand, according to “Thermal Comfort” defined by The American Society of Heating, Refrigerating, and Air-Conditioning Engineers (ASHRAE)^33^, human beings have to maintain their core temperature within a very narrow range to ensure optimal cellular and molecular function, and either overheating or overcooling can induce a major challenge to our survival. It is reported that for human skin, the comfort zone is between 30 and 34 °C^34^. Thus, for a wearable sensor that is required to attach on human skin, increasing temperature caused by heat may evoke an extreme uncomfortableness, and even hazards to human health. Because of the low thermal conductivity of human skin^35^, heat accumulation on the interfaces between sensors and human skin may occur. In this respect, materials with good thermal insulation are indispensable.

Nanocomposites composed of insulating polymer matrix and inorganic fillers are usually prepared. Herein, the role of the insulating polymer is to acquire mechanical stability, while the inorganic fillers (e.g., metal^36^, carbon materials^37,38^, and ceramics^39^) provide high thermal conductivity^40,41^. Among these inorganic fillers, boron nitride (BN) is considered as one of the most well-known candidates for thermal dissipation materials^41–44^. With extremely high aspect ratio and outstanding thermal transport properties, a low loading of boron nitride nanosheets (BNNSs) can help to obtain desired thermal conductivity without sacrificing elastomeric properties of the nanocomposites^45^. Besides, a wide bandgap (~5.9 eV) of boron nitride makes it an excellent electrical insulating property^46,47^, demonstrating a strong potential for packaging electronic devices and electric equipment that require rapid heat dissipating. Recently, we developed polymer/BNNS nanocomposite films to improve thermal conductivity using electrospun fibers. The as-spun nanofibrous arrays are capable of regulating the orientation and spatial distribution of BNNSs in the nanocomposites, leading to a remarkable thermal dissipation^48,49^. In contrast, electrospun fibrous membrane usually has a high porosity and tunable pore sizes^50^, allowing penetration and retainment of certain volumes of gases. This special architecture is an ideal candidate for thermal insulation^51^. Although remarkable achievements have been made in each field, less attention has been paid to heat dissipation of stretchable electronics. In particular, to the best of our knowledge, high-performance wearable and stretchable electronics with excellent thermal management have not been reported yet.

In this paper, we present a highly stretchable, breathable, and biocompatible strain sensor with advanced thermal management capability. This sensor has a good stretchability of more than 100%, excellent electrical properties, including high gauge factor of 35.7, and long-term durability and repeatability for more than 5000 cycles. Because a conductive nanonetwork of graphene nanoribbons (GNRs) was formed on the surfaces of electrospun thermoplastic polyurethane (TPU) fibrous membrane, precisely sensing human motion has been achieved, and it is further proved by the training of elite dragon boat paddlers. Furthermore, the nanocomposited TPU martrix filled with close-contacted BNNSs (termed as TPU-BNNSs) ensures rapid dissipation of thermal heat to environments, while the thermal insulation of electrospun TPU fibrous membrane makes it appropriate for skin-attachable electronics. The interfacial thermal conductance between the TPU-BNNS film and air is also simulated and calculated for further understanding the thermal dissipation mechanism of the strain sensor. As a result, a 242% enhanced thermal conductivity and subsequent 32% drop of the real-time saturated temperature of the sensor are achieved. In particular, for the first time, dynamic operational temperature fluctuation of stretchable electronics under repeating stretching–releasing processes has been in situ tested. During continuous stretching–releasing process between 0 and 100% strain for more than 30 cycles, the surface temperature of the strain sensor reveals an outstanding thermal stability, and the equilibrium temperature change is only within 3.5 °C. Finally, cytotoxicity test confirms that BNNSs and GNRs were tightly restricted in the nanocomposites, and there was no damage to human health. Our results are supposed to create new possibilities for the next generation of wearable devices, particularly where the extreme thermal stresses are inevitable.

## Results

### The preparation and structure of the strain sensor

**Fig. 1:**
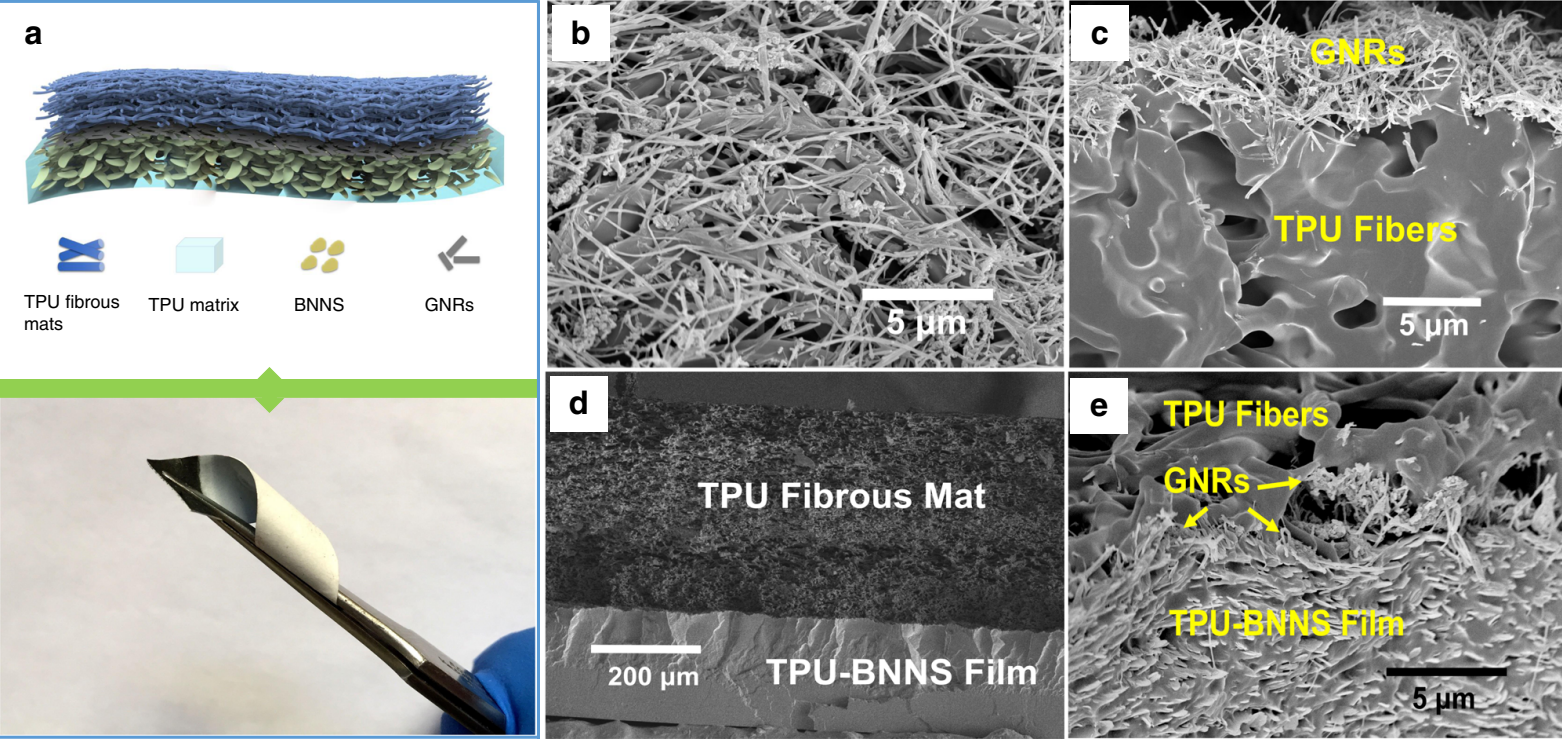
Structure schematic. **a** Sketch map and photograph of the stretchable sensor. **b** Top-view and (**c**) cross-sectional view of the GNRs deposited on the electrospun fibrous mats. **d** Cross-sectional view of the strain sensors. **e** Enlarged cross-sectional view of the interlayer between as-spun TPU fibrous mats and the TPU-BNNS film.

The schematics for the fabrication of highly sensitive, thermal conductive, and stretchable strain sensor and the photograph of the sensor are demonstrated in Supplementary Fig. 1 (see “Methods” for details of the fabrication steps). After vacuum filtration deposition, GNRs lodged on the surface of electrospun TPU fibrous membrane forming an electrical conductive nanonetwork. Then the TPU fibrous membrane with GNRs was stuck with the damp-dried TPU-BNNS film with the side where GNRs were deposited. The polymer matrix is based on TPU, TPU-BNNS film, and TPU fibrous membrane can closely integrate together, and GNR nanonetwork was sandwiched. Finally, two Cu foils were attached on both naked ends of the fibrous membrane, and the strain sensor was fabricated. Hence, a typical sensor has three layers, including electrospun TPU fibrous mats, GNR conductive nanonetwork, and the casted TPU-BNNS film, as shown in Fig. 1a. From Supplementary Fig. 2, one can see that the side of the electrospun TPU fibers is white (bottom) because the porosity of the as-spun fibrous mats causes diffuse reflection of most of visible light^52^, and the side of pure TPU film is black (middle), which is the color of GNRs due to the transparency of TPU film. When covered with TPU-BNNS film, it looks light gray (top). The GNRs have a quasi one-dimensional (1D) structure (150 nm in width, 4 μm in length), and the thickness of about 3 nm was measured by atomic force microscope (AFM) and side-view TEM of individual GNR, respectively, as shown in Supplementary Figs. 3, 4. The large aspect ratio of GNRs guarantees that they can be intercepted by the TPU nanofibers during vacuum filtration. As shown in Fig. 1b, c, most of the GNRs are deposited on the surface of TPU fibrous membrane, leading to a conductive percolating nanonetwork by entangling with each other^53^, while very few GNRs fall into the interspaces of the as-spun fibers. The BNNSs were exfoliated from commercially available hexagonal boron nitride, and showed a flat morphology with an average lateral size of 1–2 μm (Supplementary Fig. 5) and a thickness of 3 nm (Supplementary Fig. 6), consistent with our previous works^48,49,54^. After casting, the BNNSs are homogeneously dispersed, and well overlapped in the TPU matrix, as shown in Supplementary Fig. 7. The large contact area between BNNSs is important for the dissipation of heat, because they are responsible for the reduction of the thermal contact resistance when heat transfers through the nanocomposites. After covering the fibrous membrane on the surface of the casted TPU-BNNS film, it is found that the gap between the two layers diminished, resulting in a two-layered nanocomposite with the thickness of about 530 μm, including an ~345-μm electrospun fibrous membrane and an ~185-μm TPU-BNNS film, as shown in Fig. 1d. It is because that both the electrospun fibers and the as-casted film are based on TPU that renders a high intermiscibility of the two layers. In this case, GNRs were tightly sandwiched between the two TPU layers, leading to a compacted contact between BNNSs and GNRs, facilitating the thermal transport by forming integral thermal pathways, as shown in Fig. 1e. On the other hand, the electrospun TPU fibrous membrane with porous structure (Supplementary Fig. 8) has a suppressed thermal conductivity, which can help prevent the skin from burning damage when the device is attached on human skin as a wearable device.

### Basic performance of the strain sensor

The loading content of active materials plays a crucial role in determining the device performance. The contents of BNNSs in the casting TPU-BNNS film were chosen as 25 wt%, 30 wt%, and 35 wt%, respectively. For comparison, a pure TPU film was casted using the same experimental condition. With increasing the proportion of BNNSs, the stretchability of the TPU-BNNS film drops accordingly, which is attributed to the fact that the inorganic filler concentration in the matrix can affect the mechanical properties of the nanocomposite. However, after covering the eletrospun TPU fibers, the strain sensor can still tolerate a large tensile strain above 300%, as shown in Supplementary Fig. 9, illustrating the excellent mechanical properties of the strain sensor.

The sensing performance of the stretchable sensors is dependent on the loading concentration of GNRs. Here the vacuum-filtrated GNRs on the surface of the as-spun TPU fibrous mats were selected at 25 μg cm^−2^, 50 μg cm^−2^, and 75 μg cm^−2^, respectively. As shown in Supplementary Fig. 10, under a continuous strain from 0 to 100% with a fixed voltage of 5 V, the electrical property of the sample with 75 μg cm^−2^ GNRs shows an almost unchanged electrical conductivity from 0 to 100% strain, because too much electrically active materials lead to a stable conductivity under a large strain, and this low sensitivity is not suitable for strain sensors. In contrast, the normalized resistance (*R*/*R*_0_) with 25 μg cm^−2^ GNRs changes mildly under small strain less than 40%. However, it increases drastically after the strain of 60%, and a flat platform occurred. That is because the interconnecting GNRs detached under the larger stain, leading to nonconductivity of the nanocomposite. Only the normalized resistance of the sample with 50 μg cm^−2^ GNRs demonstrates a mild increase from 0 to 100% strain, illustrating the adaptability for high stretchable strain sensors.

With the rapid development of modern electronics, in particular with increasing power density and miniaturization of electronic devices, effective heat dissipation of such devices has become a critical limiting factor, because the generated undesirable heat may be concentrated in a specific area in the devices and causes the thermal failure. In an individual device, heat from an electronic component can disturb the nearby ones^55^. To evaluate the heat-dissipating characteristics of the stretchable strain sensors, thermal conductivity of the samples, including the TPU-BNNS film with BNNS contents of 25 wt%, 30 wt%, and 35 wt%, the pure TPU film, and the whole strain sensor, was measured and compared. The thermal conductivity (*K*) can be termed as

1 $$K = \rho \times C_p \times D,$$

where *ρ* is the density of the composite, *C*_*p*_ is the specific heat, and *D* is the thermal diffusivity^40^.

**Fig. 2:**
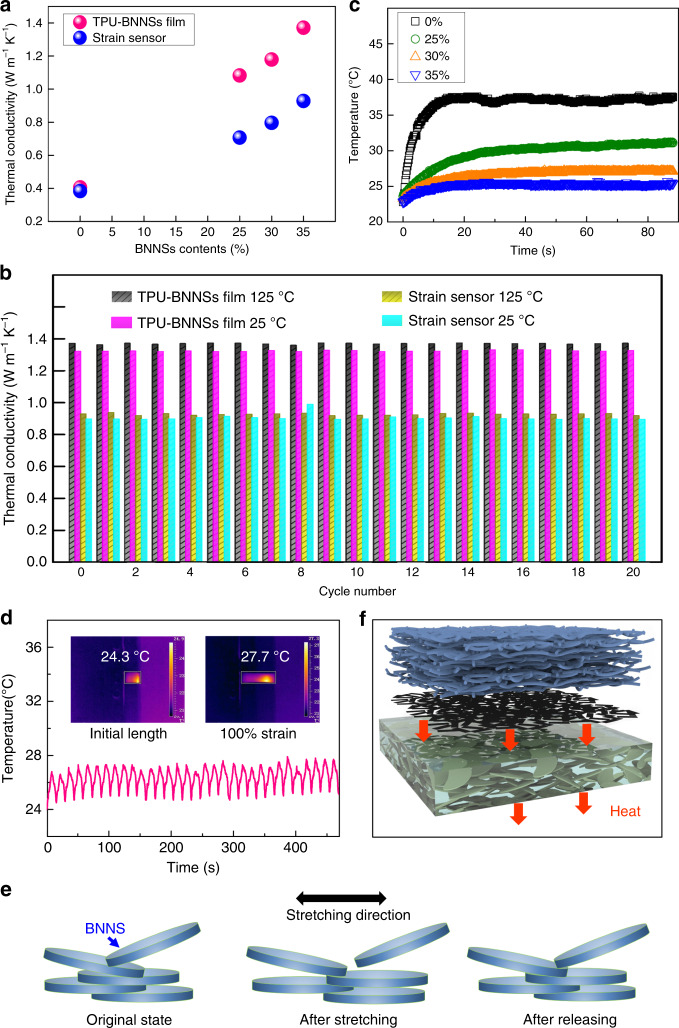
Thermal properties of the stretchable strain sensor. **a** Thermal conductivity of the TPU-BNNS film and the strain sensor. **b** Thermal conductivity of the TPU-BNNS film with 35 wt% BNNSs and the strain sensor with 35 wt% BNNSs and 50 μg cm^−2^ GNRs upon multiple heating and cooling cycles alternating between 25 and 125 °C, respectively. **c** Saturated temperatures of the samples with different BNNS loading. **d** Fluctuation range of the saturated temperature of the strain sensor under more than 30-times cycling between initial length and 100% strain to demonstrate good stability. **e** Schematic diagram of the change in thermal conductive pathways during stretching–releasing process. **f** Schematic diagram of the heat flux of the strain sensor. (*Note that the temperature of initial length in (**d**) is a little lower than the saturated temperatures in (**c**), because the clamps of the tensile platform are made from stainless steel, facilitating the thermal dissipation).

Thermal conductivity of the composite is dependent on the proportion of nanofillers that are embedded in the polymer matrix, because different contents of components and structures, the density, specific heat, and thermal diffusivity, are different accordingly. Based on the values of *ρ*, *C*_*p*_, and *D* (Supplementary Tables 1, 2), the thermal conductivity of the TPU-BNNS films and the sensors can be calculated. As shown in Fig. 2a, at 23 °C, all of the thermal conductivity of the TPU-BNNS films is higher than that of the pure TPU film in the through-plane direction. In particular, at 35 wt% loading of BNNSs, the thermal conductivity reaches 1.37 W m^−1^ K^−1^, more than 337% enhancement compared with that of pure TPU-casted film (0.406 W m^−1^ K^−1^). The thermal conductivity of the as-spun TPU fibrous mats and the GNR–TPU fibrous mats (GNR content of 50 μg cm^−2^) is only 0.0769 and 0.0949 W m^−1^ K^−1^, respectively. These two low thermal conductivities mean thermal insulativity. It is the high porosity of the electrospun fibrous mats that restricts the heat dissipation, resulting in a thermal insulativity on the fibrous side. The porous structure is befitting for function as a skin protector. After tightly sandwiching 50 μg cm^−2^ GNRs between the as-spun TPU fibers and the TPU-BNNS film, the thermal conductivity of the strain sensors has also been evaluated (blue dots in Fig. 2a). It can be seen that the thermal conductivity of the strain sensors is lower than that of the casted TPU-BNNS film, because the thermal insulativity of the porous TPU fibrous membrane hinders the thermal conductivity of the whole device to some extent. The thermal conductivity of the sample containing 35 wt% BNNSs and 50 μg cm^−2^ GNRs is of 0.928 W m^−1^ K^−1^, 2.42 times compared with the sample with 0 BNNSs and 50 μg cm^−2^ GNRs (0.383 W m^−1^ K^−1^). The thermal conductivity of the strain sensors increases with increasing the loading of BNNSs, due to the excellent thermal conductivity of both GRNs and BNNSs. The synergistic or coupling effects^56–58^ between GNRs and BNNSs can cause a rapid transmission of heat to the ambient environment, to maintain the lifetime and reliability of the devices. Figure 2b illustrates the thermal conductivity variation of the TPU-BNNS film with 35 wt% BNNSs and the strain sensor with 35 wt% BNNSs and 50 μg cm^−2^ GNRs upon multiple heating and cooling cycles alternating between 25 and 125 °C, respectively. The thermal conductivity demonstrated a slight fluctuation during 20 cycles, suggesting stable heat conduction capability of the nanocomposites in this temperature range. These enhanced thermal properties were validated by the surface temperature of the strain sensors when a voltage of 30 V is applied on both electrodes. In this course, Joule heating generated from GNRs and dissipated through the TPU-BNNS layer can be captured by an IR camera. As shown in Fig. 2c and Supplementary Movies 1–4, temperatures of all the samples increased at the beginning, and then reached the saturated temperatures within a very short time, and the range of temperature rise was in an inverse ratio to the loading of BNNSs. The saturated temperature of the samples with 35 wt% loading of BNNSs and 50 μg cm^−2^ of GNRs was about 25.3 °C, while that of the sample-covered pure TPU was 37.2 °C, much higher than the former. Moreover, for the first time, the change in the operational temperature of the strain sensor under repeating stretching–releasing processes has been measured. Interestingly, it is found that during this continuous stretching–releasing process for more than 30 cycles between 0 and 100% strain, the surface temperature of the strain sensor (35 wt% BNNSs and 50 μg cm^−2^ GNRs) demonstrated a good thermal stability, as the fluctuation of the equilibrium temperature was within 3.5 °C (Fig. 2d and Supplementary Movie 5). It can be ascribed that thermal conductivity of polymer/filler composites is dependent on the thermal conductive pathways, and the contact areas between BNNSs contribute to the reduction of thermal resistance and enhancement of thermally conductive pathways in the nanocomposites. As illustrated in Fig. 2e, after casting, the BNNSs are homogeneously dispersed, and well overlap in the TPU matrix. Under stretching, some contact areas between BNNSs decreased, and even some BNNSs are separated. It results in the absence of some thermal conductive pathways, and an increasing of operational temperature. After releasing to the original length, some missing thermal conductive pathways recovered because of the elasticity of TPU, and the temperature dropped compared with the stretching state. Although the temperature is slightly increasing than that of the original state due to the friction between BNNSs, making it difficult to keep the same contact area as the original state, the BNNSs can still recover the most to their initial stages, thus largely keeping the original temperature of the sensor (Fig. 2d).

Interfacial thermal conductance (denoted as *G*), which can be defined as the ratio of heat flux to temperature drop across the interface of two components, is another crucial parameter for designing devices and equipment where temperature and thermal stress are of concern^59^. For thin films, the temperature drop resulted from the bonding strength and material difference. To further understand the thermal dissipation mechanism of our strain sensor, we simulate the interfacial thermal conductance between the TPU-BNNS film and air. As shown in Fig. 2f, at the steady state, the heat energy per unit time generated from electricity is denoted by *Q*_e_. The heat energy flows through the TPU-BNNS layer of thickness *h* (185 μm), and the heat flux inside the layer can be given by $J_{\mathrm{l}} = K\frac{{T_{\mathrm{H}} - T_{\mathrm{L}}}}{h}$, where *K* is the thermal conductivity, *T*_H_ is the temperature of the heat source (GNRs on TPU fibrous mat without BNNSs/TPU or TPU encapsulation, 38.6 °C, as shown in Supplementary Fig. 11, and *T*_L_ is the temperature at the surface (25.3 °C)). The heat flux at the surface is given by $J_{\mathrm{s}} = G(T_{\mathrm{L}} - T_{\mathrm{a}})$, where *G* is the interfacial thermal conductance between the strain sensor and air and *T*_a_ is the environment temperature (23 °C). The heat flux per unit surface area continuity requires $Q_{\mathrm{e}} = J_{\mathrm{l}} = J_{\mathrm{s}}$, i.e.,

2 $$Q_{\mathrm{e}} = K\frac{{T_{\mathrm{H}} - T_{\mathrm{L}}}}{h} = G\left( {T_{\mathrm{L}} - T_{\mathrm{a}}} \right).$$

The value of *G* is then determined by

3 $$G = \frac{{Q_{\mathrm{e}}}}{{T_{\mathrm{L}} - T_{\mathrm{a}}}} = \frac{{K\frac{{T_{\mathrm{H}} - T_{\mathrm{L}}}}{h}}}{{T_{\mathrm{L}} - T_{\mathrm{a}}}} = \frac{{K(T_{\mathrm{H}} - T_{\mathrm{L}})}}{{h(T_{\mathrm{L}} - T_{\mathrm{a}})}}.$$

By calculating, the interfacial thermal conductance is 2.9 × 10^4^ W m^−2^ K^−1^. This high value means that there is much heat flux penetrattion through the TPU-BNNS film at a certain area and time, revealing the excellent thermal dissipation of our device.

### Electromechanical performance and human motion monitoring

**Fig. 3:**
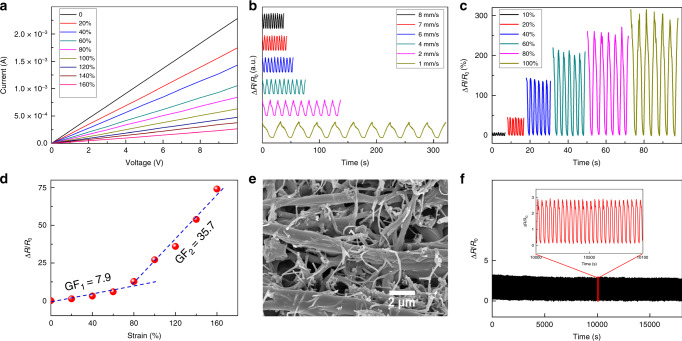
Electromechanical properties of the stretchable strain sensor. **a** *I*–*V* curves of the stretchable strain sensor under various strains. **b** Relative change in resistance versus time under strain of 100% at a strain frequency of 1, 2, 4, 6, 7, and 8 mm s^−1^, respectively. **c** Sensor response under various external strains, including 10%, 20%, 40%, 60%, 80%, and 100%. **d** Relative resistance change of the stretchable strain sensor under different applied strains. **e** SEM image to illustrate the GNRs on the surface of TPU fibers during stretching. **f** Cyclic test of the sensor at 100% for more than 5000 cycles, and the enlarged view of the marked region, showing excellent stability and repeatability.

We measured the current versus voltage (*I–V*) characteristics under diverse static strains first. As shown in Fig. 3a, from the initial length to a strain of 160%, all the *I*–*V* curves reveal linear relationships, and the slope of these *I*–*V* curves decreases with increasing the applied external strain, due to the increase in resistance under strain. To further test the electromechanical performance of the strain sensor, dynamic cycling strains are proposed. The strain sensor was characterized under diverse stretching rates at a fixed strain of 100% by testing the resistance variation

4 $${\mathrm{\Delta }}R/R_0 = \left( {R - R_0} \right)/R_0,$$

where *R*_0_ is the initial resistance and *R* is the real-time resistance during the tensile process, respectively. As shown in Fig. 3b, the value of Δ*R*/*R*_0_ increased during the stretching process and recovered when the tensile stress was withdrawn. The symmetrical output signals illustrate that the resistance recovered as quickly as in the loading cycles, indicating the structural stability of the strain sensor. Furthermore, the maximum resistance variation under different frequencies keeps steady, revealing an excellent independence on the test frequency^60^. Next, Δ*R*/*R*_0_ under different dynamic strain cycles has been investigated under strains from 10 to 100% (Fig. 3c). From the results, one can see that the maximum Δ*R*/*R*_0_ is in direct proportion to the applied strain. Apart from the linearity, sensitivity is another important factor to characterize the performance of the strain sensor, which is defined as gauge factor (GF) = Δ*R*/(*R*_0_·*ε*), here *ε* is the applied strain. By calculating, when the strain is lower than 60%, GF = 7.9, and it reaches 35.7 when the applied strain is higher than 60%, as shown in Fig. 3d. The reason is attributed to the structure change of the conductive network for electron transport between GNRs. After vacuum filtration, certain amounts of GNRs deposited on the surfaces of randomly distributed TPU fibrous membrane, forming a conductive nanonetwork by entangling each other. Under small strains, with the fiber turning aligned, GNRs on individual TPU fibers detached, resulting in the rise of resistance. Other GNRs sticking to the surfaces of TPU fibers (Fig. 3e) turned align accordingly, as shown in Supplementary Fig. 12, leading to a direct, fast charge transfer. These two contrary mechanisms make the resistance increase slowly, and it means a small GF (~7.9). When a large external strain of more than 60% is adopted, the close connection among the adjacent GNRs stuck to the surfaces of TPU fibers will be separated, and the charge transport is restricted, leading to a sharp increase in resistance of the strain sensor, and in this case, the big GF = 35.7 appeared. However, because the TPU fibers with a long length exhibit outstanding mechanical properties, they can be highly stretched without deformation occurring on GNRs, which helps the long-term stability and reliability for the device. By increasing the tensile stress, the number of the connecting points separate between GNRs increases gradually; thus, a greater sensitivity has been achieved. Finally, by withdrawing the applied strain, the sensor is released to the original state, and the GNRs go back to connect again, and the resistance returns to the initial value. The long-term performance of reliability and stability of our strain sensor is also proved by more than 5000 cycles of dynamic stretching–releasing between 0 and 100% strain, as shown in Fig. 3f. The maximum values of the signals are corresponding to those in Fig. 3c, indicating the merits of our stretchable strain sensor. As shown in Supplementary Fig. 13, after these cycles, no obvious structure separation between TPU-BNNS film and the as-spun mats has been observed. Interestingly, most of the fibers adjacent to GNRs melt due to the accumulated heat during operation. We think it can be attributed to the fact that both the matrix of casting film and electrospun fibers are from TPU, which render a high intermiscibility of the two layers. In particular, due to the energy-absorbing capacity and the large thermal conductivity of GNRs, the energy from input electricity is able to effectively transfer into the TPU matrix, and can enable TPU chains at the interface to be self-healed^61^. In addition, the as-spun fibrous membrane has a low thermal conductivity; thus, the accumulated heat cannot dissipate from this side easily. These results also illustrate the superiority of our strain sensor.

**Fig. 4:**
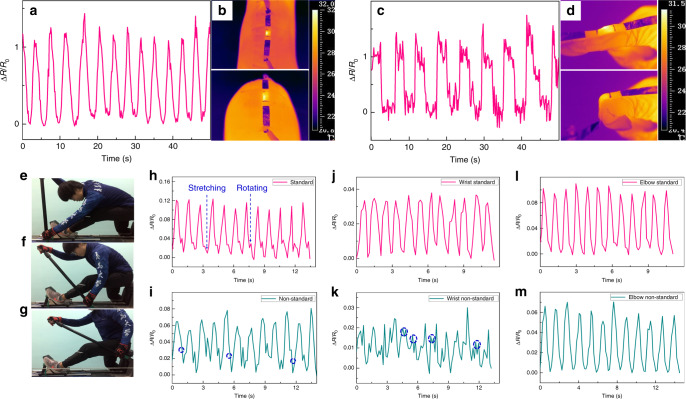
Human motion monitoring tests. Resistance change of the strain sensor by fixing the sensor (**a**) on the knee and **c** on the index finger, and (**b**) and **(d)** illustrate the stability of the strain sensor even when the joints were flexed or extended. **e**–**g** Three key places when the blade of the paddle was dug into the water and pulled it back. Resistance change of the strain sensor for dragon boat paddler training: standard states when fixed on the (**h**) shoulder, (**j**) wrist, and (**l**) elbow, and their nonstandard counterparts are shown in (**i**), (**k**), and (**m**), respectively.

As a proof of concept, we fabricated the sensors for real-time monitoring of human motion. Resistance variation for repeatedly bending–relaxing of different body joints, such as the finger and knee joints, was tested. In order to avoid slipping of the mounted sensor from the body joints, the strain sensor was attached to human joints by a surgical tape. Figure 4a shows the resistance change of the strain sensor by fixing the sensor on the knee. One can see that the resistance variation was in accordance with knee movements. Then, the sensor was applied on the index finger to detect more complicated motion. From Fig. 4c, it can be observed that resistance signals of the strain sensor were precise and repeatable during the periodic finger bending–relaxing process, and the slight trembling of the finger can also be measured. In both cases of the bending/relaxation motion, the value of Δ*R*/*R*_0_ reached a peak more than 1 and nearly recovered to its original value, indicating the high sensitivity of the strain sensor. Moreover, thanks to the tailored thermal conductivity of the strain sensor, a reliable heat transfer can be acquired even when the joints were flexed or extended, as shown in Fig. 4b, d.

In dragon boat racing, the paddlers engage their upper body to dig the blade of the paddle into the water and pull it back using shoulder as the axis (Fig. 4e–g), involving repetitive overhead arm abduction and flexion and then extension, as well as the trunk flexion and rotation, extension, and derotation^62^. Considering that the injuries are mainly of the sprain or strain of the upper body, particularly shoulder joint is the most paddling-related injury-prone (60%)^62,63^, we expect that the difference between standard and nonstandard training action can be distinguished by the strain sensor via the resistance signal. To this end, female elite dragon boat paddlers of Qingdao University who had won the third place in 2019 China dragon boat tournament finals participated in this experiment. Figure 4h shows resistance signal change of a standard training process; when the strain sensor is attached on the shoulder of a paddler, it can be seen that in each cycle, two peaks appear, which is because of the stretching and rotation of the shoulder joint. However, after 3-min training, some peaks of shoulder rotation vanished (blue circles in Fig. 4i), which is attributed to the unconformable fatigue of the shoulder. Therefore, appropriate relaxation is needed. Interestingly, the opposite phenomenon occurs when the strain sensor was fixed on the wrist. It is because usually the paddler needs to bend their wrist to achieve an equivalent range of the paddle, when they do not have enough power; therefore, multipeaks of bending emerged after 3 min of normal training, as shown in Fig. 4j, k. In both cases, the value of Δ*R*/*R*_0_ of nonstandard states is smaller than that of the standard states because of the fatigue after training, which can also be observed from the resistance signal of the elbow, as shown in Fig. 4l, m.

### Breathability and biocompatibility of the strain sensor

**Fig. 5:**
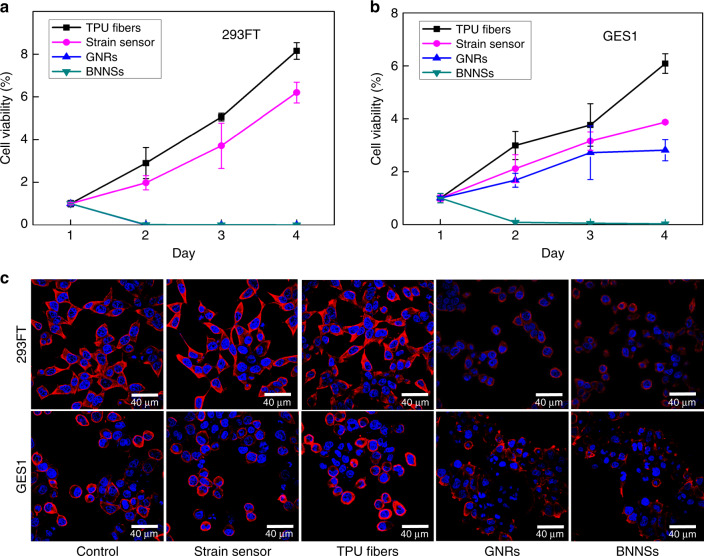
Biocompatibility of the strain sensor. Cell-proliferation assay shows that (**a**) 293FT and (**b**) GES1 cells exposed to DMEM extracts of the electrospun TPU fibers, and strain sensor, have significantly increased viability compared with those exposed to GNRs and BNNSs. Data are normalized with day 1 and represented as means ± SD. Error bar: S.D. (*n* ≥ 3). Experiments were repeated three times. Unpaired *t* tests were used to compare the difference between the two groups. *Significant relative to the control or the wild-type group, *p* < 0.05, **p < 0.01, ***p < 0.001. n.s., no statistical significance. **c** Confocal microscope image of 293FT and GES1 cells after exposure to the original DMEM (control) or DMEM extracts of the electrospun TPU fibers, strain sensor, GNRs, and BNNSs for 24 h. Cells exposed to GNRs and BNNS extracts show injured actin cytoskeleton (red) and DNA (blue).

As we know, for wearable electronics, breathability and biocompatibility are of significance because of the safety requirements of wearable electronics^64,65^, To evaluate the breathability, a gas permeability test was carried out. In this course, the strain sensor was covered on the opening of graduated cylinders containing water (in order to increase contrast, some blue ink was added into the water) on the side of electrospun TPU fibrous mats by a weight, and the sample was placed in a vacuum oven at room temperature and 35% relative humidity. The loss of water can be observed by the scale on the tube. For comparison, a casted TPU-BNNS film was operated in the same condition. After 7 days of test, it is found that the residual water of the tube with the strain sensor is much less than that with TPU-BNNS film, as shown in Supplementary Fig. 14. In this case, the process of gas permeation occurs in the layer of electrospun TPU fibrous mats, rather than the entire strain sensor. Although the gas and/or water molecules from bottom can be blocked by the top dense TPU-BNNS film, they are able to transmit along the in-plane direction of the fibrous membrane due to the high porosity (Supplementary Fig. 8). Regardless of water loss due to the poor tightness in each tube, the high degree of vapor permeability exhibits the breathability of the strain sensor. In addition, cytotoxicity tests were carried out to evaluate the safety of the strain sensor due to the importance of biocompatibility for direct human skin-attachable applications. For this reason, GNRs, BNNSs, electrospun TPU fibers, and the strain sensor were first incubated in Dulbecco’s modified eagle medium (DMEM) for 3 days. Then human embryonic kidney cells (293FT cells) and human gastric epithelial cell line (GES1 cells) were, respectively, seeded in DMEM extract from strain sensor, TPU fibers, GNRs, and BNNSs. From Fig. 5a, b, it can be observed that 293FT and GES1 cells exposed to DMEM from active nanofillers (particular BNNSs) showed significantly decreased viability, whereas those exposed to extract from the electrospun TPU fibers, and strain sensor, remained healthy, for 4 days of culture. The confocal microscope images also confirmed the biocompatibility of the strain sensor from the comparison of actin skeleton and DNA, as shown in Fig. 5c. These results show that although GNRs and BNNSs may be toxic to some living cells^66–68^, the construction of the strain sensor can tightly restrict them in the nanocomposites and protect them from leaching.

## Discussion

In summary, we report a high-performance strain sensor with advanced thermal management. The strain sensor contains TPU as matrix, and BNNSs and GNRs for thermal and electrical conductivities, and exhibit tailored thermal property. The high stretchability and sensitivity, and long-term durability of electrical properties make it an ideal candidate for precisely monitoring human motion. In particular, the as-casted TPU-BNNS film leads to an enhanced thermal conductivity, helping rapid heat transmission to the environments, while the porous electrospun fibrous membrane layer results in a thermal insulation, functioning as the skin protector. In addition, a high interfacial thermal conductance of 2.9 × 10^4^ W m^−2^ K^−1^ between the TPU-BNNS film and air has been performed, and the dynamic operational temperature fluctuation of the stretchable electronics under repeating stretching–releasing processes has been investigated for the first time. The change in equilibrium temperature within 3.5 °C for more than 30 cycles reveals the outstanding thermal stability of the stretchable strain sensor. Finally, the cytotoxicity test confirms that active nanofillers can be tightly restricted in the nanocomposites and protected from leaching, which is crucial for skin-attachable devices. We hope that our work can create new opportunities for the thermal management of next-generation wearable and stretchable electronics.

## Methods

### Exfoliation of h-BN

The liquid-phase exfoliation of hexagonal boron nitride (h-BN) was carried out according to our previous work. About 3.5 g of boron nitride powders were added into a 200-mL mixture solvent of isopropanol and deionized water (1:1 by weight). The solution was sonicated in a FS-450N model sonication bath (Shanghai Sheng Xi Ultrasonic Instrument Co., LTD) for 4 h with a frequency of 20 kHz. The resulting dispersions were centrifuged using TGL-15B super centrifuge (Shanghai Mei Ying Pu Instrument Manufacturing Co., LTD, China) at 4000 rpm for 10 min to remove nonexfoliated h-BN. The supernatants were collected and centrifuged at 9000 rpm for 30 min to collect the exfoliated BNNSs.

### Electrospun TPU fibrous membrane

First, dimethylformamide (DMF) and tetrahydrofuran (THF) (1:1 by weight) were mixed as the solvent. Then, TPU powders were dissolved in this solvent with concentration of 18 wt%, followed by continuous stirring for 5 h, until it turned into a homogeneous electrospinning precursor solution. For electrospinning, a rotating drum was adopted as the collector to get nanofibrous mats with uniform thickness. The solution was loaded in a syringe (5 mL) with a stainless-steel spinneret, which was connected to the positive electrode of a high-voltage dc power supply (Tianjin Dongwen High Voltage Power Supply Co. Ltd, Tianjin, China), whereas the negative electrode of the high-voltage dc power supply was connected to the rotating drum. The applied voltage was about 12 kV, and the work distance between the spinneret and the collector was about 12 cm. All the samples were electrospun for 2 h at room temperature with ambient humidity from 20 to 50% RH.

### Preparation of TPU-BNNS films

BNNSs were first dispersed in a mixture of DMF and THF (1:1 by weight), followed by ultrasonication for 1 h and stirring for 2 h. Then TPU powders were dissolved in this complex (the concentration was 20 wt%) by continuous stirring for 5 h. Finally, the resulting gel-like liquid was casted onto a glass plate through a doctor blade, to obtain TPU-BNNS films.

### Fabrication of the stretchable strain sensors

First, a vacuum filtration deposition method was adopted. In this course, a piece of as-spun TPU fibrous scaffold was used as the filtration membrane. As GNRs dispersed in deionized water were filtered through the membrane by a sucking pump, the DI water was able to pass through the pores among the electrospun fibers, while GNRs became lodged. After vacuum filtration, the fibrous scaffold with GNRs was put into the 80 °C vacuum oven for drying completely. When the TPU-BNNS film on the glass plate was damp-dry, a rectangular piece of sample with the size of 1 cm × 2 cm was cut from the TPU-BNNS film, and the as-prepared fibrous mats with GNRs were covered on it with the side where GNRs were deposited, followed by a 70 °C vacuum drying for 12 h to evaporate the residue solvents. Finally, the sample was peeled off from the glass plate, and two Cu foils were attached on both naked ends of the fibrous membrane, and the strain sensor was fabricated.

### Cell-proliferation assay

Cell-proliferation assay was performed according to the protocol of the CellTiter-Lumi™ Plus Luminescent Cell Viability Assay Kit (Beyotime, Shanghai, China). Briefly, strain sensor, TPU fibers, GNRs, and BNNSs were first incubated in DMEM for 3 days. Then 293FT cells and GES1 cells were, respectively, seeded in triplicate in 96-well plates at a density of 4 × 10^3^ cells per well in 100 μL of DMEM extract from strain sensor, TPU fibers, GNRs, and BNNSs. An equal volume of CellTiter-Lumi™ Plus Reagent was added to the wells and mixed for 2 min at room temperature on an orbital shaker to induce cell lysis. After an additional 10-min incubation at room temperature, the luminescent signals were measured.

### Cell-morphology analysis

293FT and GES1 cells were seeded in a 35-mm glass-bottom dish with ~70% confluency and cultured for 24 h. Subsequently, the original DMEM was replaced by an extract of the strain sensor, TPU fibers, GNRs, and BNNSs for another 24 h. The cells were fixed with 4% paraformaldehyde in 1 × PBS for 30 min and permeabilized with 0.1% Triton X-100 in 1 × PBS for 5 min. After washing thoroughly with PBS buffer, cells were blocked with PBS containing BSA (5%) for 1 h and then incubated with a-Tubulin (1:2000, Sigma) for 1 h. After washing thoroughly with PBS buffer, the cells were incubated with DAPI (1:1,000, Millipore) for 5 min.

### Characterization

The morphology and the microstructure of the TPU, BNNS, GNRs, and strain sensor were characterized by a field-emission SEM (Nova Nano SEM 450, FEI, USA). The morphology of the samples was also observed by a TEM (JEM-2010, JEOL, Japan). The optical photographs of the samples were taken via a digital camera (A6000, Sony). The electrical properties were measured using Keithley 6485 high-resistance meter system, and the strain–stress curves of the samples were obtained by a dynamical mechanical analyzer (Q-800, TA Scientific). Thermal conductivity (*K*) of the TPU-BNNS film and the strain sensor was measured through the laser-flash technique (LFA 467 HT HyperFlash, NanoFlash, Netzsch). Thermal conductivity *K* (W m^−1^ K^−1^) was calculated as a multiplication of density (*ρ*, g cm^−1^), specific heat (*C*_*p*_, J g^−1^ K^−1^), and thermal diffusivity (*D*, mm^2^ s^−1^). Namely, *K* = *ρ* × *C*_*p*_ × *D*. The density was assessed by the water-displacement method; the specific heat and thermal diffusivity were measured by an LFA 467. Thermal conductivity of the electrospun TPU fibrous membrane and GNR–TPU fibrous mats was measured by a Thermo Labo II (KES-F7, KATO Tech Co., Japan). Surface temperature of the samples was captured using an infrared thermograph (FOTRIC-226-2, China). Before we measured the surface temperature of each sample using an infrared thermograph, a precalibration has to be adopted as follows: a water-based whiteboard pen with known emissivity (Chenguang MG-2160, China, the emissivity is 0.95) was used to evenly smear on the surface of the sample under test, and then by adjusting the emissivity of the IR camera, until the surface temperature without the daub is the same or close to the daub surface temperature, the emissivity is the correct emissivity of the sample. The morphology of the cells was visualized by Olympus confocal microscope (FV3000, Japan).

## Supplementary information

Supplementary Information

Description of Additional Supplementary Files

Supplementary Movie 1

Supplementary Movie 2

Supplementary Movie 3

Supplementary Movie 4

Supplementary Movie 5

## Data Availability

The data that support the findings of this study are available from the corresponding author upon request.
