# Supplementary material for: A high performance wearable strain sensor with advanced thermal management for motion monitoring
Source: Nat Commun. 2020 Jul 15;11:3530. doi: 10.1038/s41467-020-17301-6 (PMC7363829; doi:10.1038/s41467-020-17301-6)
Supplement: Supplementary file 1 — Supplementary Information [file 41467_2020_17301_MOESM1_ESM.pdf]

Supplementary Information

A High Performance Wearable Strain Sensor with Advanced Thermal Management for Motion Monitoring

Tan, et al.

Supplementary Figures

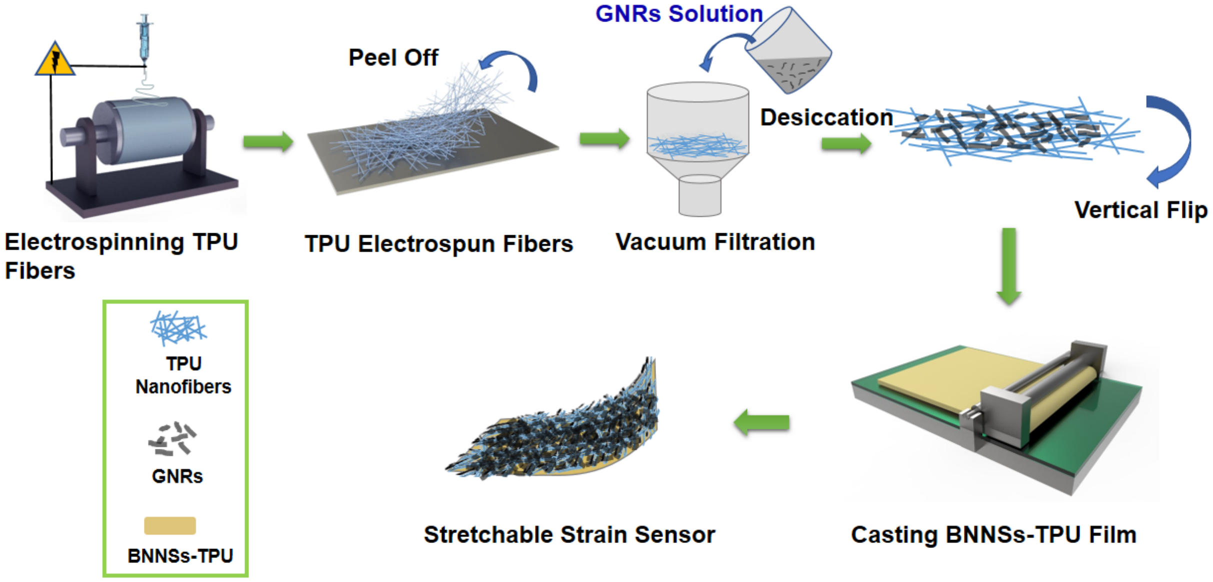

Supplementary Figure 1. Scheme of the preparation process of the stretchable strain sensor. The process mainly includes electrospinning, vacuunm filtration, casting, and stick.

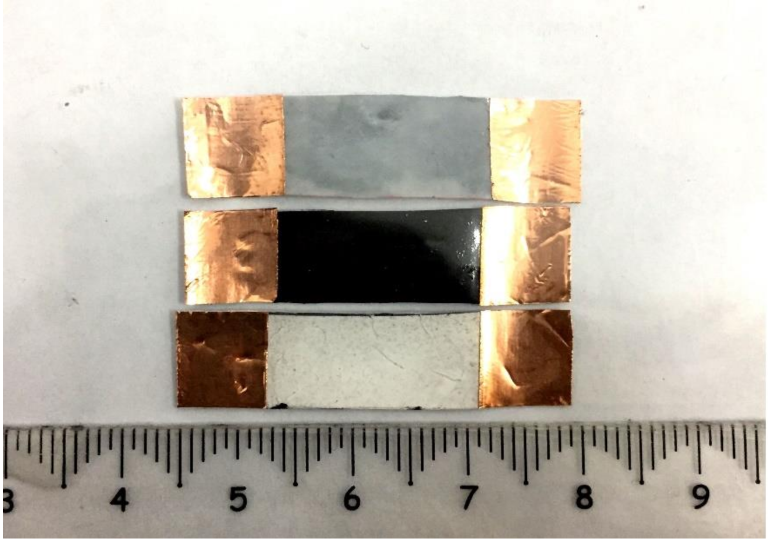

Supplementary Figure 2. Photograph of the stretchable sensor. Bottom: the side of electrospun TPU fibers, middle: the side of pure TPU film, top: the side of TPU-BNNSs film.

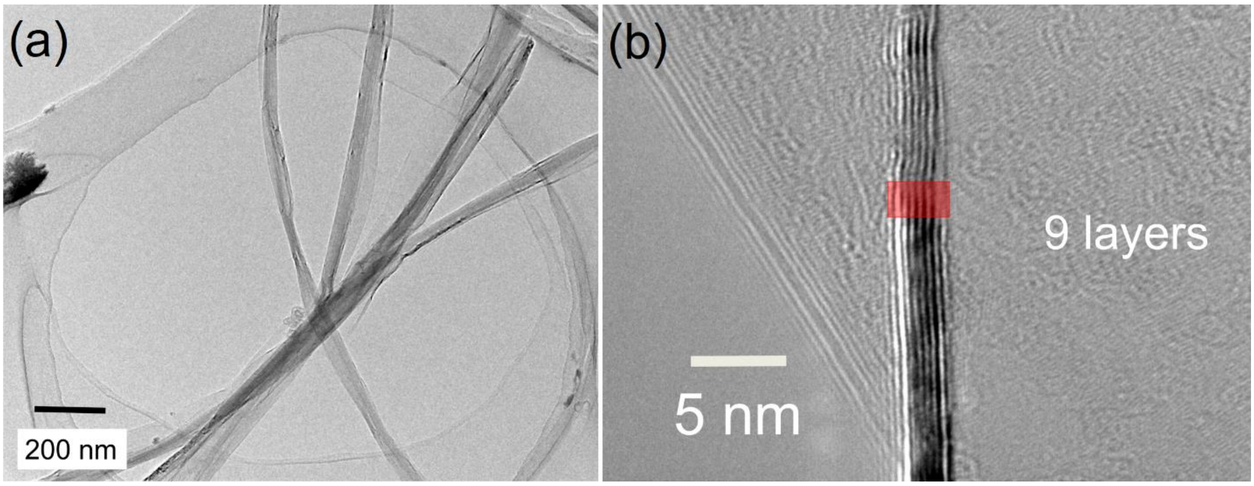

Supplementary Figure 3. TEM images of GNRs. The GNRs have a quasi one-dimensional (1D) structure. **b** the thickness of individual GNR.

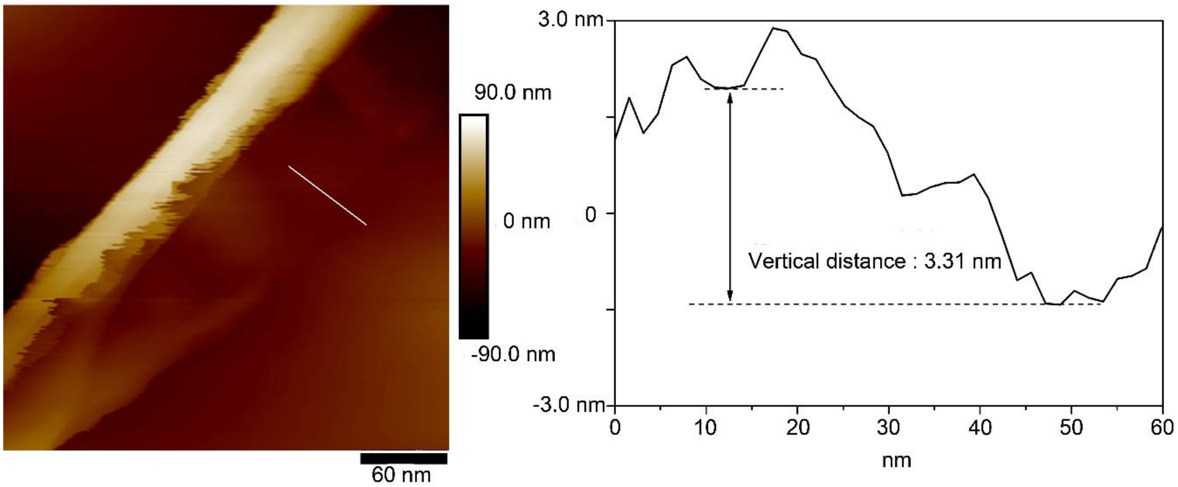

Supplementary Figure 4. AFM results of GNR and its corresponding vertical distance. The GNR has a thickness about 3 nm.

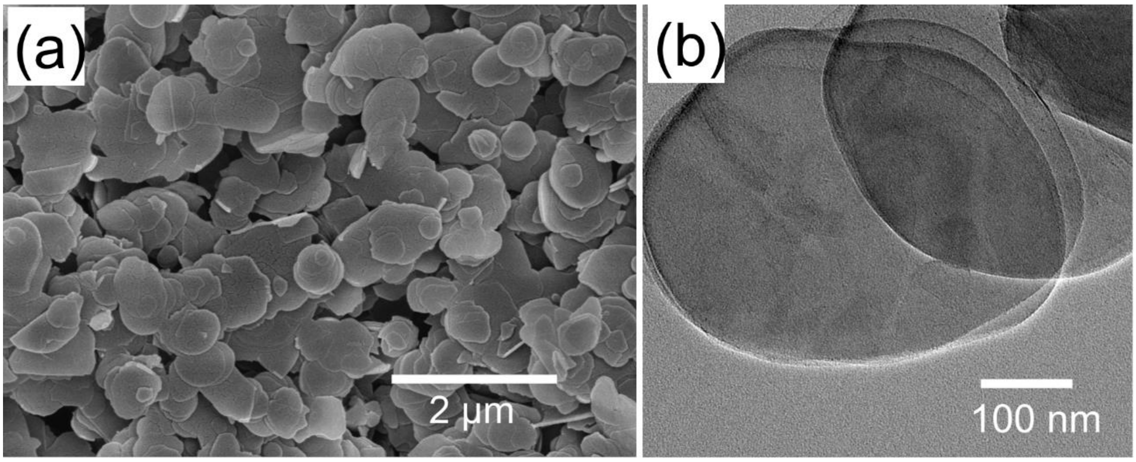

Supplementary Figure 5. SEM and TEM images of the as-exfoliated BNNSs. BNNS has a flat morphology with an average lateral size of 1-2 μm. **a** SEM image, and **b** TEM image.

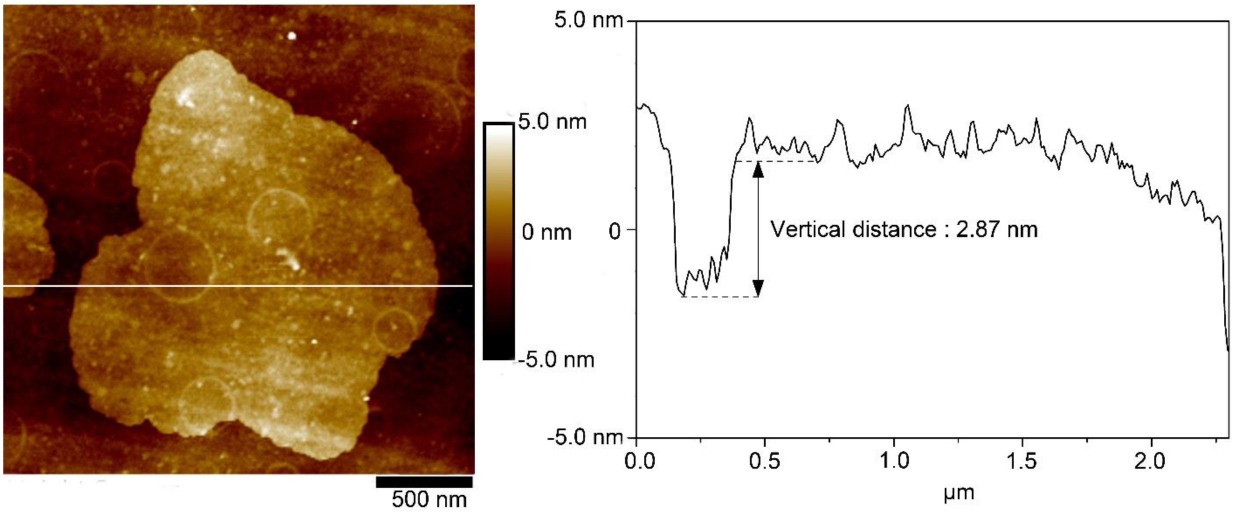

Supplementary Figure 6. AFM results of BNNS and its corresponding vertical distance. BNNS has a thickness of about 3 nm

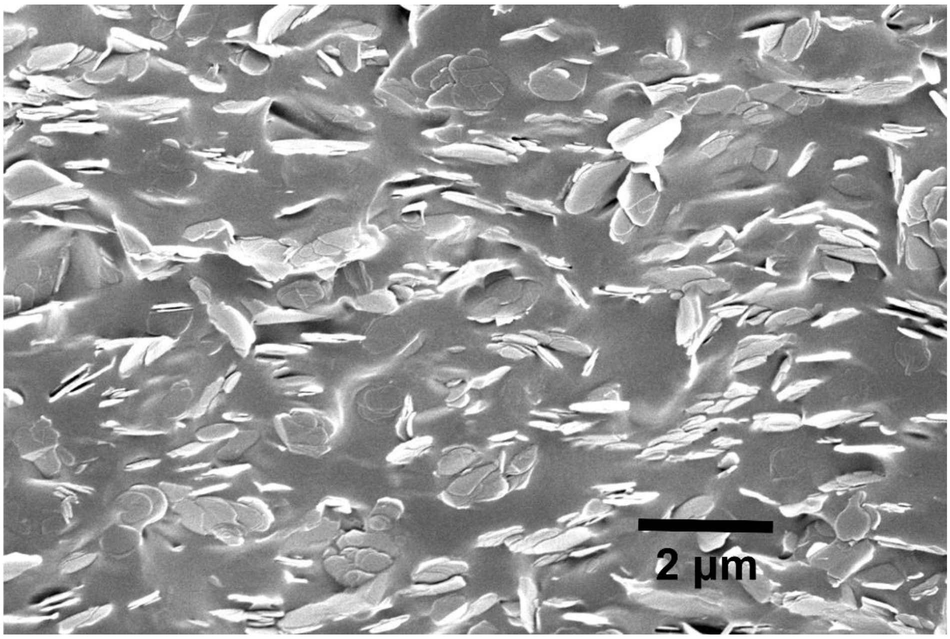

Supplementary Figure 7. SEM image of fractured surfaces of TPU-BNNSs layer. The BNNSs are homogeneously dispersed, and well overlapped in the TPU matrix.

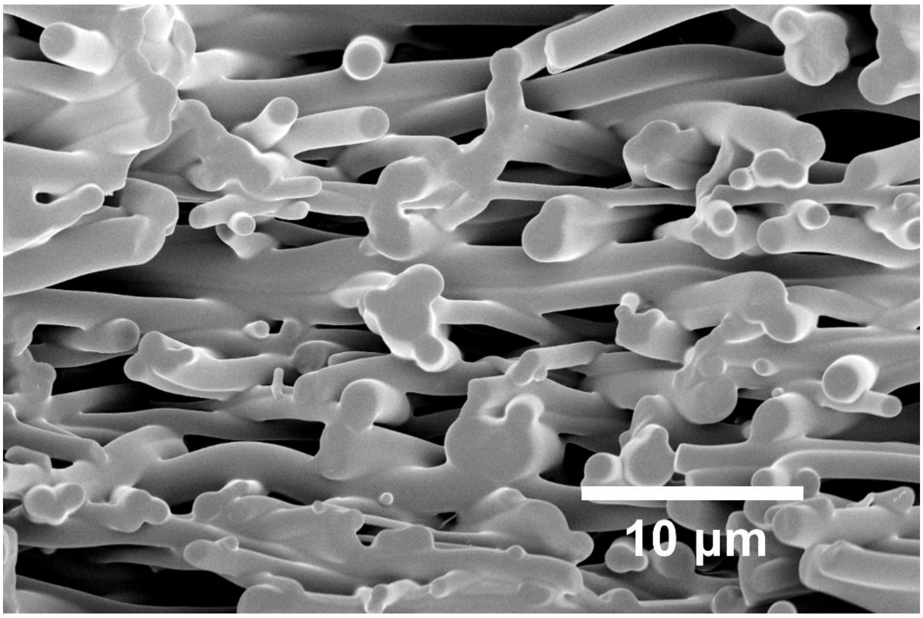

Supplementary Figure 8. Cross-sectional view of the porous fibrous mats. The electrospun TPU fibrous membrane with porous structure has a suppressed thermal conductivity, which can help prevent the skin from burning damage when the device is attached on human skin as a wearable device.

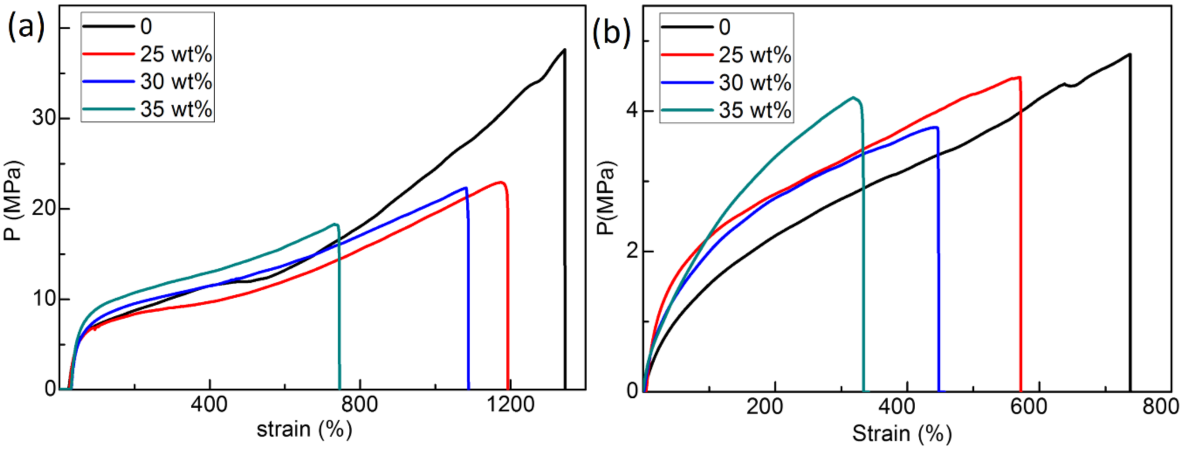

Supplementary Figure 9. Strain-stress curves of the samples. a TPU-BNNSs film and **b** strain sensor with BNNSs concentration from 0 to 35 wt %, respectively.

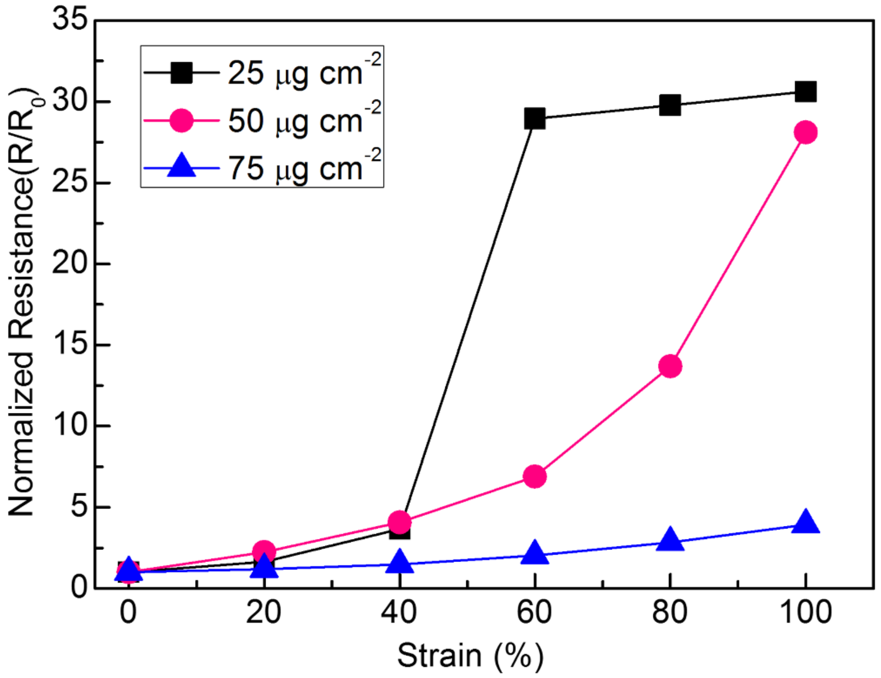

Supplementary Figure 10. Normalized resistance of the samples. The sample contains 25 μg cm^{-2}, 50 μg cm^{-2}, and 75 μg cm^{-2} GNRs under a continuous strain from 0 to 100%, respectively.

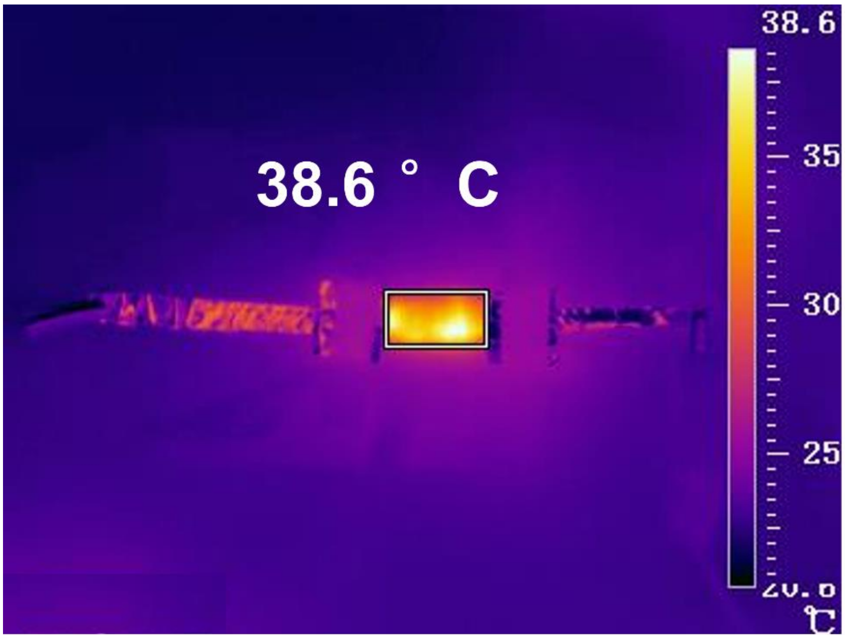

Supplementary Figure 11. Surface temperature of GNRs without encapsulation. The temperature is higher than that with encapsulation.

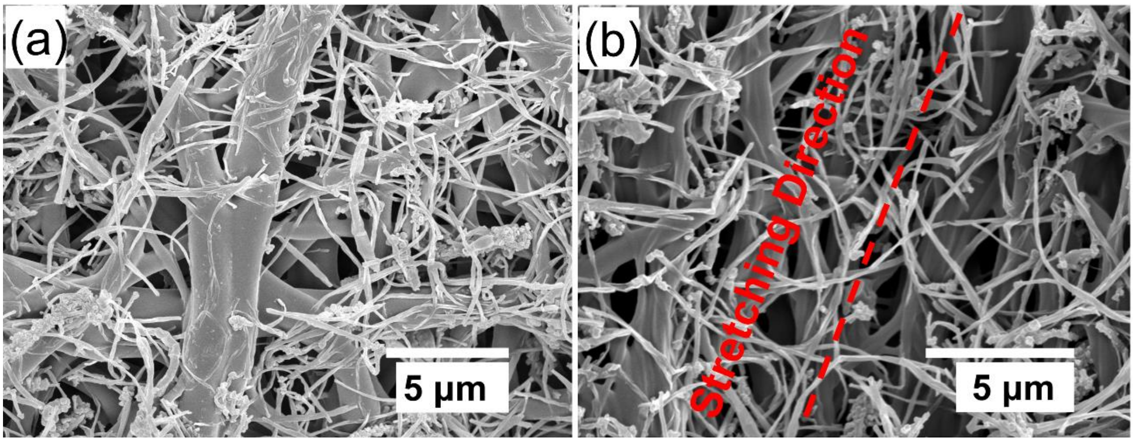

Supplementary Figure 12. SEM images of GNRs on electrospinning TPU fibrous membrane. **a** before and **b** after stretching.

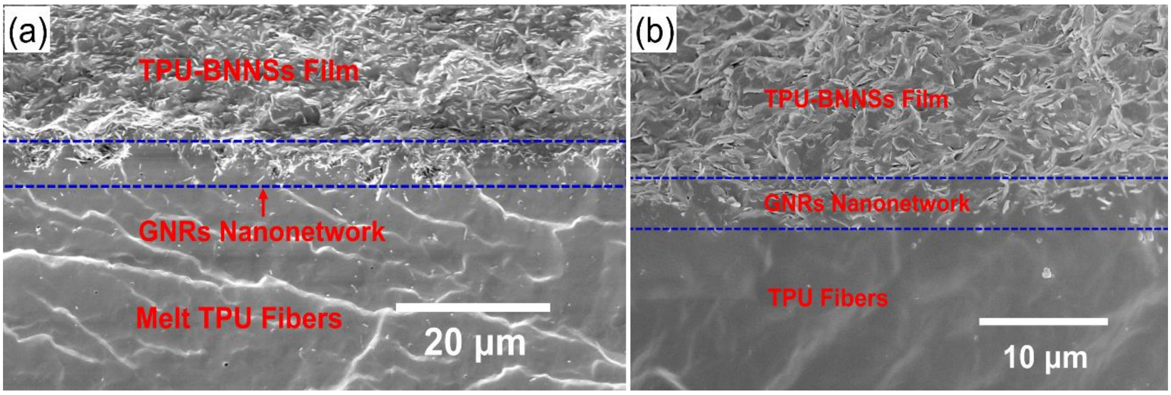

Supplementary Figure 13. Cross-section SEM image of the strain sensor after 5000 cycles. One can see that no obvious structure separation of the strain sensor has been observed.

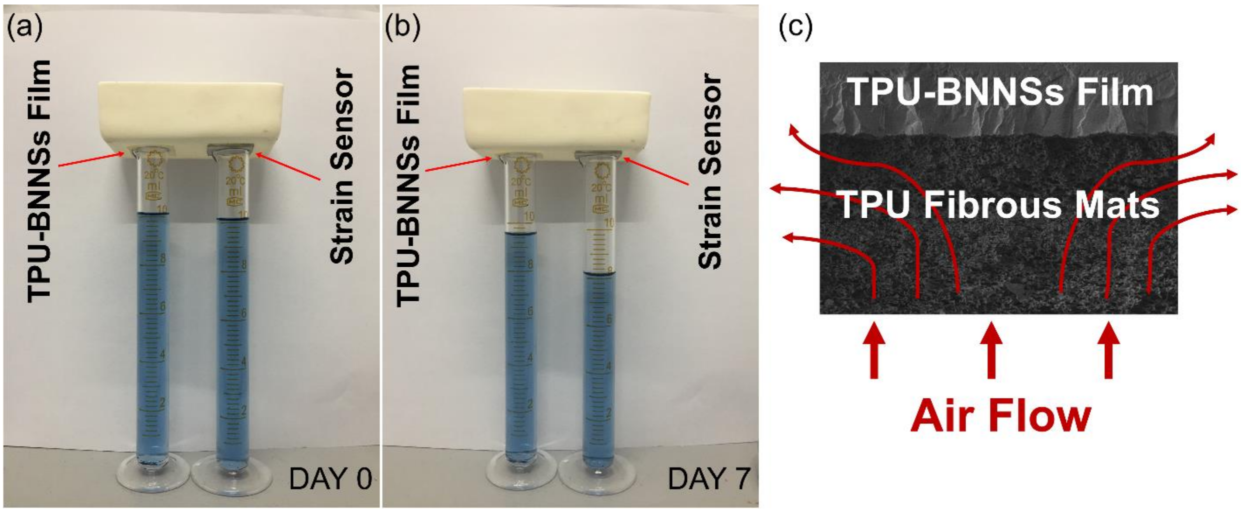

Supplementary Figure 14. Gas permeability test to evaluate breathability of the strain sensor. **a** Day 0 and **b** day 7. **c** Schematic diagram of the process of gas permeation in the strain sensor.

Supplementary Tables

Supplementary Table 1. Details for calculating thermal conductivity of TPU-BNNSs film. Density, Specific heat, thermal diffusivity, and therefore thermal conductivity of the as-casted TPU-BNNSs film with different contents of BNNSs

**BNNSs Contents**	**Density ($g\ cm^{-1}$)**	**Specific Heat ($J\ g^{-1}\ K^{-1}$)**	**Thermal Diffusivity ($mm^2\ s^{-1}$)**	**Thermal Conductivity ($W\ m^{-1}\ K^{-1}$)**
**0**	1.2180	1.7283	0.1931	0.4064
**25 wt%**	1.3559	1.7126	0.4658	1.0816
**30 wt%**	1.3966	1.7107	0.4935	1.1791
**35 wt%**	1.4172	1.6892	0.57234	1.3701

Supplementary Table 2. Details for calculating thermal conductivity of the strain sensor. Density, Specific heat, thermal diffusivity, and therefore thermal conductivity of the strain sensor with different contents of BNNSs.

**BNNSs Contents**	**Density ($g\ cm^{-1}$)**	**Specific Heat ($J\ g^{-1}\ K^{-1}$)**	**Thermal Diffusivity ($mm^2\ s^{-1}$)**	**Thermal Conductivity ($W\ m^{-1}\ K^{-1}$)**
**0**	1.1726	1.7095	0.1912	0.3833
**25 wt%**	1.2519	1.6901	0.3338	0.7063
**30 wt%**	1.2963	1.6853	0.3649	0.7972
**35 wt%**	1.3230	1.6832	0.4169	0.9284
