# Supplementary material for: A high performance wearable strain sensor with advanced thermal management for motion monitoring
Source: Nat Commun. 2020 Jul 15;11:3530. doi: 10.1038/s41467-020-17301-6 (PMC7363829; doi:10.1038/s41467-020-17301-6)
Supplement: Supplementary file 2 — Description of Additional Supplementary Files [file 41467_2020_17301_MOESM2_ESM.pdf]

Description of Additional Supplementary Files

File Name: Supplementary Movie 1
Description: Saturated temperatures of the sample with 0 BNNSs.

File Name: Supplementary Movie 2
Description: Saturated temperatures of the sample with 25 wt% BNNSs.

File Name: Supplementary Movie 3
Description: Saturated temperatures of the sample with 30 wt% BNNSs.

File Name: Supplementary Movie 4
Description: Saturated temperatures of the sample with 35 wt% BNNSs.

File Name: Supplementary Movie 5
Description: Dynamic operational temperature fluctuation of stretchable strain sensor under repeating stretching-releasing processes
